# Enacting Inner Speech on the Academic Stage. A Dialogical Review on Fossa, P. (Ed.). (2022). *New Perspectives on Inner Speech*. Springer

**DOI:** 10.1007/s12124-022-09739-8

**Published:** 2023-01-25

**Authors:** Markéta Machková

**Affiliations:** 1 Institute for Psychology and Education, University of Neuchâtel, Neuchâtel, Switzerland; 2 Department of Authorial Creativity and Pedagogy, Academy of Performing Arts in Prague, Prague, Czechia

**Keywords:** Inner Speech, Phenomenology, Play, Dialogical Acting, Performing Arts, Public Solitude

## Abstract

The recently published Springer Brief in cultural psychology presents theoretical and empirical advances on inner speech. The editor Pablo Fossa suggests viewing inner speech as a private area to *remember, play and dream*, rather than a mere psychological function connected to problem solving. Along the lines of this suggestion, I adopt a playful approach in order to review the volume. Rather than delivering results of an analysis, I invite us to use the academic journal platform to take part in a dialogical encounter. In the first part of this essay, I offer a transparent step-by-step process of researcher's positioning, based on *remembering and playing*. In the second part, I *dream* of research methodologies, which would allow us to explore inner speech as dynamic movements experienced by whole and dialogical beings. This experiment, in which I enact my inner speech on the academic stage, eventually lets three key-moments of Fossa's book come forward as gamechangers for future inquiries: 1. The importance of hearing one's voice in audio-diary based research, 2. the shift of attention towards experiential contexts of inner speech (such as bodily sensations or felt knowledge), and 3. the notion of *thirdness* as a meta-position, pointing at the mutual permeability of reflective and pre-reflective realms of inner speech. This performing review is inspired by a theatre-based practice called Dialogical Acting with the Inner Partners and represents an original contribution to researcher's self-reflexive positioning practices, as well as to inner speech qualitative research methodologies.

*New Perspectives on Inner Speech* (Fossa, 2022b) presents fresh ideas and methodological approaches related to a phenomenon, which has kept fascinating people interested in human psyche for ages and yet always remained a bit numinous. Over the course of its six chapters, collected from twelve authors, the volume invites us to meet recent advances in the inquiry into inner speech from both theoretical and empirical points of interest. Apart from the introductory and concluding chapters, written by Pablo Fossa, researcher at the University of Desarrollo in Chile, the main body of the book is divided into two parts – introducing first theoretical (p. 9–40), then empirical (p. 43–80) advances. The book got published within the SpringerBriefs edition, which might typically include "a presentation of core concepts that students must understand in order to make independent contributions".^1^ Along the lines of this publishing concept, and – given that I still have my one foot in students' world – I take this reviewing activity as an opportunity to try out how this volume allows me to understand the core principles related to the study of inner speech.

## Remember, Play and Dream within Academic Journals?

In the introductory chapter (p. 1–5), Fossa suggests to view inner speech as a "private area to remember, play and dream," distinguishing his volume's perspective on the phenomenon from a "more traditional" one, in which the main function attributed to inner speech in psychological research is supposed to be "problem solving." Fossa sums up Lev Vygotsky's critique of Jean Piaget's assumptions on the disappearing of egocentric speech in children and explains consequently where he sees "the main argument to link inner speech to problem-solving" (p. 1):

*If inner speech is an internalization of the child's egocentric language and egocentric speech has problem-solving as its main function, then inner speech also maintains this same function as the main feature.*

After presenting an overview of research ranging from 1974 to 2013, in which inner speech has been addressed "mainly from its role in problem-solving", Fossa shifts our attention towards more recent works, which "have defended the idea of inner speech at the service of other 'tasks'…" He explains (p. 2):

*I am referring to the internal tasks, the deepest motivations of human subjectivity. Here, memory, play and imagination play a fundamental role, which has nothing to do with solving problems, but with remembering the past, entertaining ourselves in a private space during leisure time or during unattractive activities, and in the projection of the future, whether distant or near.*

No matter how unsure readers might feel when it comes to what could possibly be meant by "the deepest motivations of human subjectivity," Fossa's invitations are worth considering – even if it were only to advance together a few miles further on "the winding road to the mysteries of inner speech" (this metaphor is used by the series editors Jaan Valsiner and Giuseppina Marsico in preface; p. v–vii). Thus, in the spirit of the lines above and following Fossa's simple finding that "(…) in the course of ordinary life, we need to listen to ourselves," (p. 84) I decided to pay attention to my own inner speech while working on this review.

*Straight away then, I noticed a thought articulating itself:*

*–Would it not be worth a try to use this essay to "remember, play and dream"? Rather than to deal with the review in terms of "problem solving"?*

*Then I sort of heard someone react:*

*–Come on, how could anyone play or dream over the course of an expert review essay for an academic journal?*

*Only to finally witness us reassuring ourselves that we have enough expertise and experience to vouch for such a public experiment:*

*–Oh dear, have we not been dedicated to research and education through performing arts? Do please admit it. We have been trained to play and actively imagine in public! And in academic contexts too!*

## A Public Experiment Inspired by Dialogical Acting

I am thus going to engage in a dialogue with the authors of the book under review, while taking seriously my inner dialogues as well. In other words: I am going to follow Fossa's views on people's use of the inner speech and "entertain myself during unattractive activities" (p. 2) –which (an unattractive activity) a review task could easily have become, had I taken it for a mere problem to be solved. While adopting this experimental approach, I will try (for what other reason would the word *essay* come from the French expression for *a try*?) to raise constructive questions, as well as to present associations on inner speech phenomena in general. All this is to trigger interest and suggest possible directions for further studies that could draw on our volume. As a researcher and teacher who has been working through a theatre-based practice called *Dialogical Acting with the Inner Partner(s)* (Machková, 2021; drawing on Slavíková, 2009 and Vyskočil, 2003), I will eventually contribute to the freshly published advances on inner speech by introducing a new perspective – the one which a performing methodological approach has to offer.

*–Wait a moment, are you saying you will present Dialogical Acting eventually?*

*–That's what I’ve just written. I want to give the readers a sense of a structure. Is there anything wrong about it?*

*–Nothing is wrong, really. You're doing great. Structure is a wonderful thing.*

*–It is. Isn't it? And everyone is having one in their academic papers!*

*–Sure, sure. It's just that, you know, you are actually presenting Dialogical Acting already. You kind of didn't wait for the end of the review. It's here. You keep interacting with your inner partner… or are there more of them? Partners. In front of your readers. Right now.*

*–Right. And you mean I did not warn them, or did I? And, omg, Fossa said this remembering, playing and dreaming should have been happening in a private space! I have to make this clear.*

Let me explain that my experimental writing here is already inspired by the discipline of Dialogical Acting with Inner Partner(s) (below as DA), which is – in its full and original form – practiced live, in an open space and within a group of people, guided by "assistant(s)". The theatre-like settings, drawing on the principles of "public solitude" of the actor and "wishing attention" of the audience, co-constitute laboratory conditions which allow one to practice, study and/or develop various inner speech phenomena in creative ways (Vyskočil, 2003):

*The basis of Dialogical Acting is the experience and the experiencing of interacting (speaking, playing) with oneself (with one's inner partner, or partners) which as a rule happens when one is alone. On reflecting, almost everyone should be able to recall the experience of talking to oneself, the experience of play on one's own, from one's own within. The point then is to study and learn how to produce similarly authentic, spontaneous, playful interaction and interplay (behaving and experiencing) in public (…).*

Unlike laboratory DA, my public solitude here is spread in time: I play as I write, while knowing this text is going to get published and will reach its audience later. But the play does not happen solely during my writing activity; this is what I mean by "taking seriously my inner dialogues": 1. I listen to myself as I read the book and write this review (nothing special; most people are familiar with listening to their inner speech from their own working grooves behind a computer). 2. Besides, I occasionally include transcriptions of my inner dialogues into this very text (indeed unusual). 3. But the crucial part of this experimental reviewing first really happens in moments, when I leave my desk in order to provide inner impulses with movement and voice. I explore images, thoughts and feelings as they become articulated through my body and in the space; and I observe their becoming too. Thus, some parts of what we as readers encounter at these lines as printed words, had once been enacted out loud and explored as psychophysical (i. e. felt, heard, lived through, played with). Let us see eventually, what it helps reveal on inner speech.

## *New Perspectives*: Finding Out How New They Really Are

Before reading the book under review, I wrote down a few notes regarding my knowledge of inner speech and my personal conceptions of it – to be able to tell afterwards how much new the *New Perspectives* really were to me and in what sense.^2^ Let me briefly report on my self-reflexive work as a researcher and as a person, to eventually present the ways I engage with our volume and the positions from which I view the topics. Let me introduce this positioning exercise as a discussion to authors of our volume's Chapter 5 – *An Experimental Phenomenological Approach to the Study of Inner Speech in Empathy* (p. 65–80). Vergara, Cea, Calderón, Troncoso and Mertínez-Pernía claim they adopted a phenomenological attitude in order to conduct interviews without unnecessary prejudgements or evaluations:

*To achieve that, before data collection, the researcher disclosed his own experiences and evaluations, in order to be open to observe the phenomenon that emerges without preconceptions (Hamilton et al.,* 2018*), "freshly, as for the first time" (Moustakas,* 1994*, pp. 34).*

The main concern "more traditional" psychologists have about phenomenological studies is that "people tend to have very strong preconceptions about their experience." In his popular talk, Charles Fernyhough, 2016 continues: "All you're really doing if you're asking people what their inner experience is like, is, you're asking about their preconceptions; what you think your experience is like, rather than what it is actually like." In our volume, it turns out as thrilling to see Chapter 5 authors experiment with phenomenological designs, which not only head beyond preconceptions of participants, but also consider those on researchers' end. Unfortunately though, apart from a reference to a study on experiencing flow while playing metal music and to Clark Moustakas' methodological guide, we do not get introduced to any neutralizing steps the researchers specifically took prior to leading interviews. I am interested in reading more on researchers' (self-)reflexive positioning practices as constitutive parts of research projects, since tuning oneself into a state out of which one looks at things "freshly, as for the first time" has been a key skill trained in the Authorial Acting program, within which I have been teaching and doing research^3^ (and learning the alchemy of finding a "zero-point" usually takes students two to three years).

Below, my reviewer's self-positioning report comes in four parts: first, playing with a dictionary definition of inner speech; second, recalling my personal experience; third, remembering academic resources. Finally, I take the advantage of my DA practice, while offering a closer look into *New Perspectives* chapters*.*

### Playing With a Dictionary Definition

To gain a bigger picture of what is commonly understood under inner speech, I made some basic search. When I looked the term up in my Mac Dictionary, I have learned that it was a *mass noun*, standing for "the silent expression of conscious thought to oneself in a coherent linguistic form."^4^ After having read the *New Perspectives* thoroughly *(-Warning: Spoiler follows!)*, I could imagine I would alternatively define inner speech e. g. this way: "the embodied emergence of felt knowledge within one's dialogical being-in-the-world in a form of sensorial imagery". By offering this provocatively contrary view I do not claim that authors of our volume meant to put the latter in the place of the former. The popular definition of inner speech is well reflected throughout their book, and gets enriched with previously sporadically opened dimensions.

*–What would you say to that: If we put the two definitions digestedly next to each other, we might get the idea about the nature of novelties brought up in the book.*

*–And sum them up for the readers this way? Sure. Would a table come in handy?*

*–Oooh, baby I love your wa-ay… You know we love tables, right?*

*–Tables look science!*

The following table presents my attempt to name some general aspects, which for the authors imply the need for more awareness of inner speech's complex nature and the urge to study it via revised and innovative methods.

**Table:** 

Inner speech definition as found in a dictionary application	Reviewer's improvised variation after reading *New Perspectives*	Reviewer's sketch of complexifying aspects described in *New Perspectives*
The silent	The embodied	Inner sp. can occur via non-articulated voice, body...
expression	emergence	Active stance vs. passive living through.
of conscious	of felt	Multiple modalities of consciousness / awareness.
thought	(felt) knowledge	Levels of articulation.
to oneself	within one's dialogical being-in-the-world	One's self is co-constituted by otherness and environment.
in a coherent	in a form of condensed	Questions of density degree and type of order.
linguistic form.	sensorial imagery.	Multiple modalities of inner speech (also pre-reflective).

As we can see, the common sense premises are enriched with dimensions, which have often been neglected in late research, partly due to the complexity they carry along. This enrichment is achieved by means of elaborations on theoretical contexts (e. g. Edmund Husserl's transcendental phenomenology, p. 9–21), methodological approaches (e. g. an experimental phenomenological approach, p. 65–80) and research designs (e. g. audio online diaries, p. 43–63).

*–What did you say again? I couldn’t hear you well, Edmund kept distracting me with his transcendences.*

*–Something is missing. I can’t really put into words what it is, wait for it, please. Hmmmm…*

*–You always miss something, don't you? Go on, tell me, if you have to.*

*–I don’t know, I’ve just had that feeling, like when you forget your keys at home or something. – (pause) – Ok, I guess it’s this: The job *New Perspectives* does, is not solely complexifying. At some places–and this is the most striking thing–the act of admitting inner speech being far more complex, seems to be opening a door to simpler and more straightforward ways to study it, you know?*

*–Do you have this lady in mind, the "Laura case" from Chapter 4?*

*–Exactly! The ease with which she, a research participant, actually discovers a precious qualitative method.*

*–Ok, let us keep the idea and develop that once we come to the performing approaches, ok?*

*–Because you think her use of voice messages in real time connects to performativity? Thrilling.*

### Recalling Personal Experience

As a next step prior to reading the book, I tried to see how I could relate to the inner speech on a more personal level. First, as a family member, appearing in a variety of roles burdened with social expectations, I thought of stressful situations, in which I have always desperately been hoping for another voice to come up – sort of speaking from "the heart" (an empathic position), and yet as if from a certain distance, to provide me with perspective. Second, as a spiritually exercising person, I realized the processes of prayer and meditation had been relying on inner speech; they could be defined as intra-personal dialogues with an imaginary/felt Other, or classified among "dialogues between human and non-human actants" (for a comprehensive summary of forms of dialogues and types of dialogicality in connection with imagination see e. g. Zittoun, 2014). And third, as a person experiencing inner episodes of "déjà-vu worlds" (which have recently been attributed a diagnostic label of *epilepsy*), I recalled that an unusual modality of consciousness has been accompanying them. Now, for the purpose of this essay, I will not elaborate on any of these personal spheres of experiences, but…

*–Why not? That could have been so much fun!*

*–Rofl indeed. But neither at the right place, nor at the right time! Just be so kind and let us save something for our final PhD authorial presentation,*^5^* would you?*

In any case, all these three spheres of experience clearly suggest possible directions for inquiries into inner speech or relate to the existing ones: The first association points at the use of inner speech as linked to roles individuals play within their social environments and it can be said that Chapter 4 of our volume touches this subject matter (*Expressiveness and Psychic Internality: The Use of Online Diaries in the Contemporary Forms of Life*, p. 43–63). The second example shifts our attention towards the relationship between inner speech and spiritual practices (for this direction see e. g. Moffett, 1982; Puchalska-Wasyl & Zarzycka, 2019), whereas the third example points at inner speech as parts of a specific human condition (which under some circumstances and in certain cultural contexts can be associated with personal health issues, be considered disease symptoms and ground medical diagnosis and treatment); in *New Perspectives*, a current discourse on the frontiers between health and disease is introduced e. g. in Chapter 5 through referencing the works of Alderson-Day and Fernyhough (e. g. Alderson-Day & Fernyhough, 2015; Alderson-Day et al., 2018). It is clearly an overall forte of the volume that it helps demystifying common perceptions of inner/private speech, which sometimes keep being reduced to somehow unsettling "hearing of voices in our heads", "talking to ourselves".

### Remembering Academic Resources

To take some distance from the personal levels of experience, I made myself remember solid literature. The socio-cultural psychologist in me wrote down the name of Lev Vygotsky whom I got to know as a founding father to the inner speech notion. As a researcher on dialogicality, I came up with William James's famous *I* and *me* distinction (James, 1890/1983) as well as with some more recent work, such as Ivana Marková on the *inner Alter* (Marková, 2006; drawing on Bakhtin et al., 1979/1986). As a philosophy reader, I recalled some Socratic thoughts from one of Plato's dialogues, which searched for a proper definition of knowledge and (as if by the way) described ways a person's opinions had been formed *in* and *by* the soul. *–And how did *New Perspectives* meet my preconceptions?* Since I knew I was about to review for the *Psychology and Cultural Developmental Science* section of SpringerBriefs,^6^ it was no surprise that I eventually read on Vygotsky's work in every chapter of the book. I also enjoyed an expert-ego-satisfaction as James and Marková appeared in Chapter 3 that has been dealing with psyche's polymorphism and liminality (p. 27, resp. 35), and Marková again in Chapter 5 in connection to "the idea that inner speech is also shaped by multiple symbolic social representations" (p. 66). Finally, I could not help feeling sympathy for the volume editor (whom I never met before) when I read a quote from Plato's *Theaetetus* in the introductory chapter.

Given his interest in the history of thought, Pablo Fossa's will to refer to pre-Vygotskian legacy is only natural. From the section *About the Editor and Contributors* we learn that Fossa is currently pursuing his second PhD, this time in philosophy, and he also co-authors Chapter 2, which connects Vygotsky with the German philosopher Edmund Husserl. In general, this will and interest imply there is a need for a historical-philosophical account of how inner speech had been addressed before psychology got divided from philosophy. This might indeed be an adventurous path to follow, however, no resources are mentioned in Chapter 1 apart from Plato and Saint Augustine; a few words from a Socrates line are quoted and Saint Augustine's *Confessions* are merely named (p 1). Since the reference to Plato is done in an unfortunate way and cannot be found in the bibliography, I allow myself to present the passage in the footnote,^7^ taken from Fowler's English translation (Platon & Fowler, 1987) that can easily be consulted online – for those who get eager to revisit ancient texts, while keeping inner speech on their minds as a key-word. Regarding Saint Augustine of Hippo: Despite the popularity of his *Confessions*, in which he makes sense of his personal life story while being extraordinarily honest and entertaining (Bragg, 2018), it seems to me, that passages relevant for inner speech are to be found elsewhere – as discovered by Matthews, 1967: "The inner man has an inner mouth and this the inner ear discerns (…)" (*On Continence*, II, 4). Or even more pertinently, in *On the Trinity*, XV, 20 (Augustine & Matthews, 2002):

*For all words, no matter in what language they may sound, are also thought in silence; and hymns run through our mind, even when the mouth of the body is silent; not only the numbers of the syllables, but also the melodies of the hymns, since they are corporeal and belong to that sense of the body called 'hearing,' are present by their own kind of incorporeal images to those who think of them, and silently turn all of them over in their minds.*

This passage – because written by a spiritually awakened person who at the same time had been practicing corporeal religious rituals and had worked hard to make sense of his own embodied and situated life story via writing – relates precisely to the distinctions and paradoxes (e. g. words vs. images, felt knowledge vs. thought) encountered by the authors in their research and presented at several places of the book.

*–See, you didn’t want to let us quote Augustine, you thought it would be too intellectual or even too religious, and in the end… Do you realize…*

*–…his writings helped us view some important dichotomies? which reappear throughout the reviewed book?*

*–Exactly. But you will have to explain what we mean. (pause) –Oh dear, I know that glimpse of yours. You are about to insert a table again, aren't you?*

Let us now benefit from the instant inspiration by Augustine's incarnate thinking, hearing and writing. Admitting that a new need has emerged and allowing it to shape the structure below, I am going to sum up some above sketched *New Perspectives* issues, as they appear under more specific names in individual chapters. My most pertinent experience of inner speech – the one lived through and observed in the DA practice – will get some attention later*.* After all, it is precisely the distinctions derived from corporeal vs. incorporeal tensions, which might help us see the potential of performing practices for inner speech inquiry.

## Can One Overcome Body-Mind Split by "Intellectual Dances"?

Fossa's distinction between "remember-play-dream" and "problem-solving" contexts of inner speech emergence is crucial for the whole volume: it sets the scene for a variety of other, more or less similar dichotomies, which researchers (Chapters 2–5) navigated in their efforts to theoretically grasp and empirically study inner speech. Let us have a look at the book's outline and the distinctions–no matter if accidentally discovered, intentionally used or just encountered as paradoxes–in specific chapters.

**Table:** 

	Chapter in *New Perspectives on Inner Speech*	Reviewer's attempt to sum up distinctions used / dichotomies encountered	
Preface	On the Borders of Inner Speech (Valsiner & Marsico, 2022)	Dancing	Intellectually
Chapter 1	Inner Speech: The Private Area to Remember, Play, and Dream (Fossa, 2022a)	Remember-Play-Dream	Problem Solving
Part I	Theoretical Advances on Inner Speech		
Chapter 2	Reflective and Pre-reflective Inner Speech (Fossa & Pacheco, 2022)	Pre-reflective	Reflective
Chapter 3	The Otherness in the Constitution of the Psyche: Arguments from Psychoanalysis and Cultural Psychology (De Luca Picione & Freda, 2022)	Sociability (Otherness)	Individuality (Identity)
Part II	Empirical Advances on Inner Speech		
Chapter 4	Expressiveness and Psychic Internality: The Use of Online Diaries in the Contemporary Forms of Life (Pinheiro et al., 2022)	Expressiveness	Internality
Chapter 5	An Experimental Phenomenological Approach to the Study of Inner Speech in Empathy: Bodily Sensations, Emotions, and Felt Knowledge as the Experiential Context of Inner Spoken Voices (Vergara et al., 2022)	Experiential Context of Inner Speech	Objectifying Settings in the Research Design
Part III	Conclusion		
Chapter 6	The Inner Other: Who Speaks When I Speak to Myself in Silence? (Fossa, 2022c)	Passive-Suffered Experiencing (Other)	Reflected-Aware Executing (Self)
		THIRDNESS (Meta-Position)	

In his concluding Chapter 6, Fossa sums up the dichotomies I have observed by following words, while coupling together chapters 3 and 4 as well as chapters 2 and 5 (p. 83):

*For De Luca Piccione and Freda, otherness plays a fundamental role in the construction of identity in particular and of the psyche in general; meanwhile, for Pinheiro, Mélo and Barros, otherness is expressed in the dialogical nature of the inner speech during the recording of online diaries.*

*(…)*

*(…) what Fossa and Pacheco present constitute the theoretical framework of what Vergara et al. show empirically, that is, a pre-reflective and pre-conceptual dimension of inner speech, non-propositional and bodily felt.*

Clearly, the authors have done their best to bring attention to body-, feelings-, expressiveness-related aspects of inner speech. Yet, despite the freshness of their advances, we read in the editors' preface, that "the well-meaning authors perform intellectual dances at the doorstep of the cabinet of curiosities of the inner speech–fearing to enter." (p. v) *–How come? –The adjective "intellectual" seems of particular importance here.* As I was reading, it occurred to me, that all the distinctions made and dichotomies described could be assigned one common denominator of a more general distinction: *yes!* the one of mind–body split, which had influenced research epistemology across disciplines, and psychology in particular–as it has been shown e. g. by Tau et al., 2022:

*Mind-body dissociation had a remarkable impact on twentieth century psychology. Although there were solid anti-reductionist research programs, it is possible, however, to recognize the prevalence of conceptual models in which the body has no participation in the explanation of cognitive and affective processes (Searle,* 2004*).*

*(…) the underlying meta-theory is that of the split, expressed through multiple dichotomic pairs–mind/body, individual/society, acquired/innate and feeling/reason, among others (…).*

Given the deep roots of the "meta-theory of the split", getting above its dense systems seems challenging even for innovative scholars. However, Fossa et al. managed to view their distinctions as two sides of same coins, rather than split disparities. They have put admirable efforts to picture the couples in a yin-yang mode, in which one side necessarily entails aspects of the other and in which dynamic and permeating movements are possible (as e. g. between the intertwined otherness and identity or pre-reflexivity and reflexivity). Moreover, a noteworthy component has been introduced, bridging the distinct couples: a meta-position called "thirdness".

*–This is fun, isn’t it? Our mission here is to defend the authors against the series editors.*

*–Veritable paladins we are! Ktož sú boží bojovníci!*

*–Ho, ho, ho, slow down there, would you? You can’t quote something just like that in your mother tongue, once you write and publish in English.*

*–But it’s a famous song and I can’t prevent it from appearing, it’s the cultural baggage I carry with me no matter where I go, and…*

*–Look. You are right indeed and it is also true that we encourage DA students and participants to act in their mother tongues. But this will be read as a written text in the end, and you need to make it accessible in the language you agreed on with the journal.*

–*I see. It reminded you of the curiosities we’ve come across in the *New Perspectives* bibliographies, right? Things like Heidegger's *Ser y tiempo* or even Kant's *Crítica de la razón pura*!*

*–Exactly. Titles that are obviously available in English translations. Don't get me wrong, you know how much I enjoyed trying to pronounce them out loud in Spanish–a language I can’t speak. And one can only agree with the series editors, that "It is remarkable that a young group of scholars, mainly located in Latin America (...) are pursuing one of the most interesting of Vygotsky's lines of inquiry." But come on…*

*–I see your point. Once the SpirngerBriefs are here to introduce students to various subject matters, it would seem reasonable to give a good example and respect the common practice of quoting and referencing too?*

*–And so should you with your fifteenth century Hussite war song!*

*–Right. But, you know what? It just appeared on its own. In a joke. And I don't feel like referencing war songs. Let us get back to the intellectual dances.*

It has to be said, that despite having remained mostly intellectual, Fossa et al. at least dared to "perform dances" at the inner speech's doorstep. In my opinion, there are at least three moments in our volume, in which the door to "the cabinet of curiosities" opens for a few inches, or even further, moments which allow us to realise there might never have been any solid door between the outer and inner, between the other and self. In the last part of this review, let us have a closer look at these key moments.

### Key Moment 1: Laura Enjoys Hearing Her Own Voice

The most fascinating thing in the study on expressiveness and psychic internality (Chapter 4) is, that it became a truly dialogical research, as one of the participants herself spontaneously contributed to it by modifying the qualitative method. Instead of writing down diary entries, she began to keep her online diary in an audio form (voice-recordings captured via a messaging application). She shortly explained she liked to hear herself and ended up regularly pursuing a practice of a dialogical speech.

*–In the conditions of "public solitude", was it not?*

Thus, in this audio-diary "practice of research" (–an expression which I appreciate as it puts weight on the very process of exploring, p. 57), what later got reflected upon and analysed as data, had previously been lived through, bodily experienced, articulated, pronounced and heard by Laura (sometimes probably also by her close others), in her more or less private environments, from her embodied situation and over the course of her everyday life. Moreover, viewed from the DA perspective, as she was recording her entries, she actually experienced a (limited) sort of "public solitude": she was aware her recordings were going to be listened to, yet not forced to interact with researchers' questions directly. Thus, she kept developing her spontaneous dialogical interactions with herself, while benefiting from the future "wishing attention" of the audience (researchers).

*–Just like you do while writing this essay!*

*–Sure, except for we're not in a covid home-confinement right now. And do hold your exclamations back for a while, would you? I'm dealing with some serious stuff here.*

This performing moment would not have any power to open doors of any cabinets, had it not been noticed, highlighted and theoretically danced around by Pinheiro, Mélo and Barros; had they not let themselves get inspired by the lively "practice of research". As Fossa points out in his conclusions, the advances made in Ch 4 appear particularly sharply, when seen against the historical-theoretical background prepared in Ch 3, which shows otherness as co-constitutive of individual psyches. It is eye-opening to realize that inner speech–as far as we understand it as a dialogical phenomenon rooted in otherness–turns out as more accessible for research inquiries! In the end, the two chapters suggest there had never been any solid door to inner speech–nothing that would as a rule remain closed, nothing that would need huge efforts to get open. Yet still, the body-mind split heritage seems to keep nourishing our "fear to enter", or at least hesitations about full enjoyment of inner speech explorations in situated, embodied, whole and dialogical beings–us, researchers inclusive.

To briefly report on the theoretical Chapter 3; De Luca Picione and Freda trace, map and rescue notions on human psyche's polymorphism, while they dutifully engage with a whole scale of resources in the history of psychology across the spectrum of its late sub-fields. Surprisingly enough, the role of the other (and thus of the social, cultural, i.e. contextual aspects of psychological life) comes forward as crucial–not only in the works of James or Vygotsky, but also in those of Freud, Jung, Stern, Hillman and others! A challenging puzzle for the readers remains, that not a single time is "inner speech" directly mentioned throughout the whole Ch 3. Nevertheless, it is brilliant in bringing together psychoanalysis and social and cultural psychologies. It provides junior researchers and students with an original theme-based overview, as well as with terminological arsenal to help grasp possible empirical findings.

### Key Moment 2: A Sensation Just… Electrifying

The experimental phenomenological study too (Ch 5) is aware of problematic aspects of asking people about their inner experience. To prevent finding out solely about people's thoughts and preconceptions, Vergara, Cea, Calderón, Troncoso and Martínez-Pernía have designed interviews focusing on bodily sensations, emotions and "felt knowledge". For me, it was a pleasure to be presented data as e. g. "I don’t know how to explain it, it was a sensation just… electrifying (…)" or "(…) and then he fell and it was like: 'aaaah'!" (p. 74). These moments allowed me to see deeper into the imagery nature of inner speech. However, given that I view the researcher-participant-stimuli interactions from the DA perspective, which is also known as "non-object acting" (acting in which we use no props, costumes, technically mediated effects, prepared texts and not even ready ideas), the methodological design stays rather rigid in my eyes–specifically in its objectifying aspects. Researchers construct laboratory conditions and expose participants to technologically mediated stimuli of empathy for pain. In comparison with the diary-based research (Ch4) this study appears depleted of the aspects of inner speech that can only be brought about through spontaneous processes. Despite looking into empathy and asking about bodily sensations, the question remains: What kind of inner speech emerges, when participants are (in an objectifying manner) exposed to sophistically constructed stimuli? (Even neuroscientists seem to have found out that different parts of brain–responsible either for producing speech, or for hearing–get activated, depending on whether inner speech occurs spontaneously, or, is artificially triggered; Fernyhough, 2016).

*–Is there not an irony? Experiential phenomenological researchers deliberately limit themselves? Deplete their design of possible naturally occurring situations and reactions?*

*–Maybe they did it for the sake of the hard scientific reliability of their research? You know, what can seem depleted to us, could possibly talk to neuropsychologists, cognitive scientists and others relying on hard empirical data and imaging technologies.*

Vergara, Cea, Calderón, Troncoso and Martínez-Pernía decided to 1) use a mixed methods design, and to 2) narrow their research question into the relationship between inner speech and empathic experiences. The work thus stands for high hope that lots of psychologists have for inter- and transdisciplinary dialogues. The authors themselves admit this aspiration (Vergara et al., 2022):

*(...) given the enactive premise that the body is both an experiential structure and a living dynamical system, future work should also include neurophysiological measures that correlate with the phenomenological ones. This view would achieve a mutually illuminating explanatory interaction between the phenomenology and neurophysiology of inner speech, as envisioned by Varela's neurophenomenology (Bitbol & Petitmengin, *2017; *Olivares et al.,* 2015; *Thompson et al.,* 2005; *Varela,* 1996*).*

### Key Moment 3: Thirdness

The theoretical elaboration on pre-reflective dimensions, which we get as a result of Fossa and Pacheco's bringing together Vygotsky's inner speech with Husserl's passive synthesis of consciousness, will for sure find its use in future studies. Chapter 1 becomes particularly vivid when Lakoff and Johnson's notion of embodied language is taken into account (p. 19; Lakoff & Johnson, 1980) however, this inspiration doesn't get as exploited as it could; the authors' claim does not convince me, that pre-reflective (or pre-verbal, less conscious, uncontrolled) components of inner speech are not shareable with others. And this is where I think practice-based research can be of immense help–to let us live through various dimensions of inner speech, and, to let us feel and witness embodied and not necessarily reflective or verbal means of sharing and communication.

In the course of my work on this essay, I took part in the *Pilot Workshop on Movement Improvisation for University Teachers* in Lausanne (for context see Kloetzer et al., 2020).^8^ There, in a small group of people, we were exploring ways to integrate performing arts knowledge and improvisation practices into academic teaching. We were learning some basic tools and playfully trying out each other's teaching designs. One of the exercises underwent was a short guided meditation, in which participants were invited to capture/picture one specific word on their minds, experience how it feels–to have the word on mind, to finally let it and observe it/feel it go through a very slow process of articulation (breathing, tuning voice, preparing mouth and face muscles, whispering it and saying it out loud). Obviously, I could not avoid linking my experience to the notion of inner speech I have been working on: I lived this body-mind awareness practice as a movement between moments of intentional actions and moments of "suffered" occurrences, while both modalities were intertwined by a third meta-position of a curious inner observer. The exercise made me realize the true key moment of Fossa's theoretical work did not consist in the term of pre-reflexive speech, but in his finding that there has been no sharp border between the reflexive and the pre-reflexive (p. 85):

*Of course, there is no sharp division between reflexivity and pre-reflexivity; on the contrary, the experience is passive-suffered, at the same time that we control and direct our cognitive processes toward the different goals or tasks of the environment. It is here that a new component emerges, along with otherness: thirdness. There is also a third position in inner speech, which transcends the self-other relationship and which observes or analyses dialogical positions.*

Just before writing this last part of the review, I came back from Zurich, where I led a DA workshop for the students (and a couple of brave teachers) of the Zurich University of the Arts.^9^ Even though I was only supposed to assist to the participants' practice and provide them with feedback, one of the students suggested we could switch roles for the few last minutes, so that I too could enjoy my embodied inner speech explorations–an experiment against rules, which I accepted with pleasure. It was my practice and the feedback I received, that made me think of how Fossa's "thirdness" would relate to the DA practice and theory. As I was enacting my inner dialogues on "the stage" and in front of "the audience", I realized that Fossa's "third position, which transcends the self-other relationship" has in DA been called "the inner spectator" (Musilová, 2018):

Public solitude *enables the student to turn her/his attention to her/his psycho-physical impulses that can become objects or themes – topics of the play. The presence of the spectators and the student's awareness of her/his existence awaken her/his inner spectator. The audience also creates an energetic field and an atmosphere, which relates through the inner partner to the student's expressivity and intensity of her/his acting. Vyskočil states that "whatever is happening on stage involving concentration, pleasure, and enjoyment, etc., is likewise […] followed in the audience. So, this is transmitted from stage to the audience. And then back to the stage again and into the play." (Roubal,* 2011*)*

Methodologically, one's "inner spectatorship" is something to be triggered, maintained and cultivated–for instance through a regular practice in the conditions of "public solitude". I am curious if and how Fossa et al. would consider a possible integration of a–let us say–"thirdness cultivation" into future research methodologies.

## Conclusion

This review, approached as a performative act, portrays the main question/suggestion I would like to add to the volume's message: What if we explored ways to inquire into inner speech in public? Not retrospectively, not only through reflecting interviews, but in the real time? During our very interactions? Through a collective, embodied and reflected living through? At conferences, in classrooms, on pages of academic journals?

In this essay, following the reviewed book's spirit, I decided to play. My experiment was inspired by Dialogical Acting, which itself has had theoretical roots in phenomenology (just as our volume), particularly in Eugen Fink's attempts towards an ontology of play. I hope for my experiment–along the following lines–that I have managed to escape the usual routines of academic life while relating to them profoundly and portraying them at the same time (Fink, 1957/2016, p. 21):

*Play "interrupts" the continuity and context of our course of life that is determined by an ultimate purpose. It withdraws in a peculiar manner from the other ways of directing one's life; it is at a distance from them. But while it appears to escape [entziehen] the standard flow of life, it relates [bezieht] to it in a manner that is particularly imbued with sense, namely, in the mode of portrayal [Darstellung].*

*–May I have one last word please?*

*–Oh, good to know you are still here! And I bet you didn't forget about the idea to quote Plato again.*

*–No, it seems pertinent. Just as a post scriptum, you know? A private message to Pablo Fossa.*

*–"Private"… ha ha. Go ahead then!*

*– Plat. Theaet. 146a: Why are you silent? I hope, Theodorus, I am not rude, through my love of discussion and my eagerness to make us converse and show ourselves friends and ready to talk to one another. (Platon & Fowler,* 1987 *)*

Note: I am grateful to Eva Slavíková, Tania Zittoun, Michaela Raisová and David Machek for their reading and precious comments, and, to Giuseppina Marsico and Jaan Valsiner for their venturesome invitation.

## Data Availability

The data I used in this article (transcriptions of enacted inner speech) are stored in my personal diaries and can be consulted upon kind request followed by the data subject enthusiastic consent.

## Footnotes

1 Quoted from https://www.springer.com/gp/authors-editors/book-authors-editors/springerbriefs.

2 This exercise, recommendable to each potential reader/unexperienced reviewer not only triggers their playful and dialogical engagement with a book, but one can also use it to check-up on their expertise. For the specific role of a reviewer, one can profit from comparing their expert views with the commonly/online available ones, as well as from becoming more aware of their biases in specific subject matters.

3 Authorial Acting is a BA/MA program, studied at the Department of Authorial Creativity and Pedagogy at the DAMU in Prague. More about the program can be found here: https://www.damu.cz/en/department/department-of-authorial-creativity-and-pedagogy/

4 Dictionary application by Apple Inc.

5 More on the performing format of "authorial presentation" can be found online: https://www.youtube.com/watch?v=ScHox2X83bQ.

6 "The series will have a clearly defined international and interdisciplinary focus hosting works on the interconnection between Cultural Psychology and other Developmental Sciences (biology, sociology, anthropology, etc.). The Series aims at integrating knowledge from many fields in a synthesis of general science of Cultural Psychology as a new science of the human being." Taken from https://www.springer.com/series/15388.

7 (Plat. *Theaet.* 189e–190a): ***Socrates.*** *Excellent. And do you define thought as I do?* ***Theaetetus.*** *How do you define it?* ***Socrates.*** *As the talk which the soul has with itself about any subjects which it considers. You must not suppose that I know this that I am declaring to you. But the soul, as the image presents itself to me, when it thinks, is merely conversing with itself, asking itself questions and answering, affirming and denying. When it has arrived at a decision, whether slowly or with a sudden bound, and is at last agreed, and is not in doubt, we call that its opinion; and so I define forming opinion as talking and opinion as talk which has been held, not with someone else, nor yet aloud, but in silence with oneself. How do you define it?* ***Theaetetus.*** *In the same way.*

8 The workshop was part of the ASCOPET project, co-organized by the Instant-Lab (EPFL) and the Institute of Psychology and Education (Univeristy of Neuchâtel). https://www.epfl.ch/labs/instantlab/ascopet/

9 Getting to know and joining their PAC (Performing Arts in Contexts) network may be of interest to readers prone to practice-based research/pedagogy. https://www.pac-now.online/
